# iTRAQ-Based Comparative Proteomic Analysis Reveals Molecular Mechanisms Underlying Wing Dimorphism of the Pea Aphid, *Acyrthosiphon pisum*

**DOI:** 10.3389/fphys.2018.01016

**Published:** 2018-08-07

**Authors:** Limei Song, Yuhao Gao, Jindong Li, Liping Ban

**Affiliations:** ^1^State Key Laboratory of Animal Nutrition, College of Animal Science and Technology, China Agricultural University, Beijing, China; ^2^Department of Grassland Science, College of Animal Science and Technology, China Agricultural University, Beijing, China; ^3^Affiliated High School of Peking University, Beijing, China

**Keywords:** wing dimorphism, Acyrthosiphon pisum, migration, Homoptera, iTRAQ, proteomics, olfactory sense

## Abstract

Wing dimorphism is a widespread phenomenon in insects with an associated trade-off between flight capability and fecundity. Despite the molecular underpinnings of phenotypic plasticity that has already been elucidated, it is still not fully understood. In this study, we focused on the differential proteomics profiles between alate and apterous morphs of the pea aphid, *Acyrthosiphon pisum* at the fourth instar nymph and adult stages, using isobaric tags for relative and absolute quantitation (iTRAQ) in a proteomic-based approach. A total of 5,116 protein groups were identified and quantified in the three biological replicates, of which 836 were differentially expressed between alate and apterous morphs. A bioinformatics analysis of differentially expressed protein groups (DEPGs) was performed based on gene ontology (GO) and Kyoto Encyclopedia of Genes and Genomes (KEGG). KEGG enrichment analysis showed that DEPGs mainly participated in energy metabolism, amino acid biosynthesis and metabolism, and signal sensing and transduction. To verify the reliability of proteomics data, the transcriptional expression of 29 candidates of differentially expressed proteins were analyzed by quantitative real-time PCR (qRT-PCR), showing that 26 genes were consistent with those at proteomic levels. In addition, differentially expressed proteins between winged and wingless morphs that were linked to olfactory sense were investigated. Quantitative real-time PCR revealed the tissue- and morph-biased expression profiles. These results suggested that olfactory sense plays a key role in wing dimorphism of aphids. The comparative proteomic analysis between alate and apterous morphs of the pea aphid provides a novel insight into wing development and dimorphism in aphids and will help facilitate our understanding of these concepts at molecular levels.

## Introduction

Phenotypic plasticity is a life history strategy of organisms, allowing them to adapt to various environmental conditions (Hall, 1999; West-Eberhard, 2003). Polyphenism is an extreme phenomenon of phenotypic plasticity in which multiple discrete phenotypes are produced by the same genotype in developing organisms in response to extrinsic factors (Nijhout, 1999, 2003). Wing polyphenism of insects has been considered to contribute to their diversity and evolutionary success (Roff, 1990; Dudley, 2002) and has evolved in numerous insect taxa, including those from the orders Coleoptera, Diptera, Heteroptera, Homoptera, Hymenoptera Lepidoptera, Orthoptera, Psocoptera, and Thysanoptera (Zera et al., 1997; Whitman and Ananthakrishnan, 2009). Wing polymorphic insects exhibit common dispersal and non-dispersal morphs (Roff, 1986; Braendle et al., 2006). Dispersal morphs of insects, with long wings and wing musculature, are capable of long-distance flight and migration to new habitats with fresh resources from deteriorated environments (Harrison, 1980; Roff, 1990), whereas short-wing or wingless morphs, without flight muscles, produce offsprings earlier and have greater reproductive output relative to dispersal morphs (Harrison, 1980; Zera et al., 1997; Simpson et al., 2011). In other words, wing dimorphism involves trade-offs between flight capability and other traits (Zera et al., 1997; Simpson et al., 2011). Wing dimorphism has been studied across a wide range of wing-polymorphic insect species, such as short-/long-winged morphs in crickets (Zhao and Zera, 2002), migratory locusts (Simpson and Sword, 2009; Tanaka and Nishide, 2012), and planthoppers (Denno et al., 1989; Xue et al., 2010), as well as wingless (apterous)/winged (alate) morphs in aphids (Brisson et al., 2007; Xu et al., 2011; Yang et al., 2014; Shang et al., 2016; Vellichirammal et al., 2016).

Wing dimorphism in aphids is associated with their complex life cycle (Brisson, 2010). Several aphid species exhibit clear differences between alate and apterous morphs. In addition to the presence or lack of wings and wing musculature, the differences can also be found in morphological, physiological, behavioral, and life cycle aspects. Alate morphs not only possess wings and flight muscles but also have more extensive sclerotization of heavier sclerotized head and thorax, more developed compound eyes, ocelli, larger numbers of secondary rhinaria on their antennae, and some species also have larger siphunculi and cauda (Kring, 1977; Miyazaki, 1987; Tsuji and Kawada, 1987a; Ishikawa and Miura, 2007) compared with apterous morphs. In addition, winged morphs are also more resistant to starvation, have a longer life, and have a more elaborate sensory system for flight navigation and for detecting host plants (Tsuji and Kawada, 1987b; Hazell et al., 2005). Olfaction plays a key role in the perception of chemical signals in insects.

The pea aphid, *Acyrthosiphon pisum* (Harris) (Homoptera: Aphididae), is an prominent sap-sucking pest in several species of legumes (Fabaceae) worldwide, including pea, clover, alfalfa, and broad bean (Blackman and Eastop, 2000), causing damage to the host plant by feeding on their phloem tissue directly as well as transmitting many viruses indirectly (Van Emden and Harrington, 2017). The pea aphid is a good study model organism with alate and apterous morphs that reflect the trade-off of dispersal and fecundity. The transition from the fourth instar winged-nymph to alate adult is the key period in aphid wing development (Brisson et al., 2010; Shang et al., 2016). The publication of the whole genome sequence of *A. pisum* provides a platform for better understanding the wing dimorphism of aphids at the molecular level (The International Aphid Genomics Consortium, 2010).

Wing dimorphism of aphids has been studied extensively to elucidate the molecular mechanism, including the analysis of the gene expression between winged and wingless adults (Brisson et al., 2007; Yang et al., 2014; Shang et al., 2016; Vellichirammal et al., 2016). However, these studies have mainly been performed at genomic and transcriptomic levels and focused on the adult stage. Proteomic analyses of wing dimorphism in insects are lacking. In recent years, advances in mass-spectrometry (MS)-based approaches for proteomics have enabled us to investigate the mechanisms of the wing dimorphism of insects at proteomic levels. Isobaric tags for relative and absolute quantitation (iTRAQ) is an isobaric labeling approach combined with liquid chromatography and tandem mass spectrometry to identify and quantify proteins (Cha et al., 2012; Ren et al., 2016) and has been increasingly used in the past few years (Brewis and Brennan, 2010; Unwin, 2010). The objectives of this study are (1) to investigate the differential protein expression profiles between alate and apterous morphs of the pea aphid at different developmental stages and (2) to investigate the potential functions of chemoreception genes in wing dimorphism of aphids. Our study provides a novel insight into the molecular mechanisms of wing dimorphism in aphids.

## Materials and methods

### Insect rearing and sample collection

The pea aphid *A. pisum* used in this study was established in 2014 from a single female adult aphid collected from an alfalfa field at China Agricultural University, Beijing, China. Aphids had been established in the laboratory for more than a year before subsequent experiments. Stock colonies were maintained on vetch seedlings (*Vicia faba* Linnaeus, 1753) in a climate-controlled environment at 20 ± 1°C with 70–75% relative humidity and a photoperiod of 16: 8h (Light:Dark). Winged morphs were induced under high-density conditions after being transferred to new host plants (Sutherland, 1969; Ishikawa et al., 2008). The impact of rearing conditions lasts over two or three generations (MacKay and Wellington, 1977). The specimens including wingless adults (AWL), wingless fourth instar nymphs (N4WL), winged adults (AW), and winged fourth instar nymphs (N4W) were collected with three replicates (200 aphids for each sample) and frozen in liquid nitrogen immediately and then stored at −80°C for future use. Tissues (antennae, heads without antennae, legs, thoraxes, abdomens, and wings) from alate adults were dissected under the microscope and individuals from each development stage of aphid (first instar nymphs, second instar nymphs, wingless third instar nymphs, wingless fourth instar nymphs, wingless adults, winged third instar nymphs, winged fourth instar nymphs, and winged adults) were collected. Samples were stored at −80°C until needed.

### Protein preparation and iTRAQ labeling

Proteins were obtained by grinding samples in liquid nitrogen, dissolved in moderate lysis buffer (7 M carbamide, 2 M thiocarbamide, 0.1% CHAPS), suspended for several seconds, followed by ultrasonication (0.5 s on, 2 s off), and then incubation at room temperature for 30 min before being centrifuged at 15,000 × g for 20 min at 4°C. The supernatant was collected and a Bradford protein assay (Sigma) was used to determine total protein concentrations (Bradford, 1976). The supernatant proteins were lyophilized and then kept at −80°C for further analysis.

Protein digestion was conducted according to the filter-aided sample preparation (FASP) procedure described in a previous study (Wiśniewski et al., 2009). In brief, for each sample, 100 μg of proteins were solubilized in 10 μl reducing reagent at 37°C for 60 min. Then 2 μl cysteine-blocking reagent was added at room temperature for 30 min followed by centrifugation at 12,000 × g for 20 min. The filters were washed with 100 μl of dissolution buffer (Applied Biosystems, Foster City, CA, USA) and centrifuged at 12,000 × g for 20 min, which was repeated three times. Proteins were then in-solution digested with trypsin (Promega) according to the protein/trypsin ratio of 50:1 at 37°C overnight. Then, the filter unit was transferred to a new tube and centrifuged at 12,000 × g for 15 min. The filtrate was collected, and the peptide concentration was estimated by ultraviolet (UV) light spectral density at 280 nm (Wiśniewski et al., 2009).

**Figure 1:**
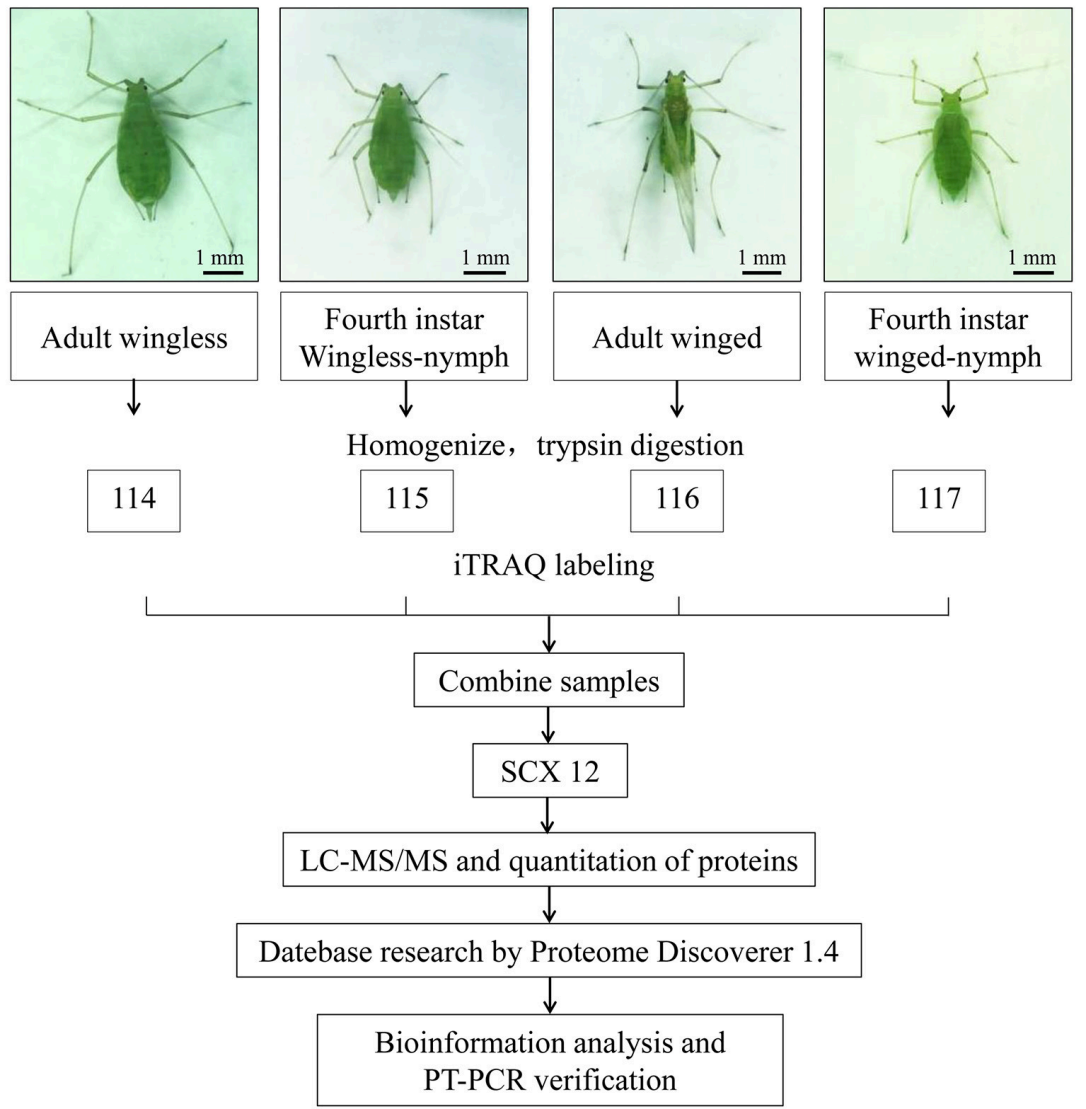
Schematics represent the experimental workflow in this study. Winged and wingless fourth instar nymphs and adults of *A. pisum* were selected, and iTRAQ-coupled 2D LCMS/MS was used to explore the proteomic differences between alate and apterous morphs. Three sets of biological replicate samples were analyzed.

According to the manufacturer's protocol, iTRAQ labeling of the peptide samples was performed using iTRAQ reagent 4-plex kits (AB Sciex Inc., MA, USA). For every development stage of the pea aphid, three biological replicates were iTRAQ labeled. The iTRAQ tags for each sample were 114, 115, 116, and 117 (AB Sciex, Foster City, USA) (Figure 1).

### Reverse-phase (RP) HPLC

The pooled iTRAQ mixtures were resuspended in buffer A (2% acetonitrile, 98% water with ammonia at pH 10), loaded onto a 4.6 × 250 mm Durashell-C18 column (150 Å, 5 μm particles, Agela), and fractionated by high performance liquid chromatography (HPLC) (RIGOL, China). Peptides were eluted using an increasing acetonitrile gradient with buffer A and buffer B (98% acetonitrile, 2% water with ammonia). The flow rate was 0.7 ml/min and the elution gradient was as follows: 5–8% buffer B for 0–5 min, 8–18% buffer B for 5–35 min, 18–32% buffer B for 35–62 min, 32–95% buffer B for 62–64 min, 95% buffer B for 64–78 min and changed to 5% buffer B within 4 min. Fractions were collected every 1 min, pooled into 12 fractions by intervals, and dried by vacuum centrifugation. All samples were stored at −80°C.

### LC-MS/MS analysis

The peptide fragments from each sample were redissolved in 2% methyl alcohol and 0.1% formic acid and then centrifuged at 13,000 rpm for 10 min. LC-MS/MS was carried out using an Easy-nLC nanoflow HPLC system connected to a Q Exactive mass spectrometer (Thermo Fisher Scientific, San Jose, CA, USA). The mass spectrometer was operated in the data-dependent mode with positive polarity at electrospray voltage of 2.0 kV. MS spectra (full scan) were acquired over a range of 350–1,800 *m/z* and resolving powers of the MS scan and MS/MS scan at 100 *m/z* were set as 70,000 and 17,500, respectively. In addition, MS automatic gain control (AGC) target was 3e6, and maximum injection time was 80 ms; MS2 AGC target was 2e4, and maximum injection time was 19 ms. Normalized collision energy (NCE) was 30% and dynamic exclusion was set to 18 s. Each sample was loaded onto Acclaim PepMap 100 C18 (2 cm × 100 μm, 5 μm C18) using an autosampler and then the sequential separation of peptides on Thermo Scientific EASY column (EASY-Spray column, 12 cm × 75 μm, C18, 3 μm) was accomplished with a gradient of buffer B (100% acetonitrile and 0.1% formic acid) at a flow rate of 350 nl/min with the following conditions: 6–9% buffer B for 0–8 min, 9–14% buffer B for 8–24 min, 14–30% buffer B for 24–60 min, 30–40% buffer B for 60–75 min, 40–95% buffer B for 75–78 min, 95% buffer B for 78–85 min, and then changed to 6% buffer B within 1 min and equilibrated for 4 min.

### Protein identification and quantification

The raw data were analyzed using Proteome Discoverer 1.4 software (Thermo Fisher Scientific) with Mascot search engine (Matrix Science, London, UK; version 2.2) to identify the proteins in a search of the protein database of *A. pisum* downloaded from the National Center for Biotechnology Information (NCBI, http://www.ncbi.nlm.nih.gov/) (download date March 7, 2016). For protein identification, search parameters were as follows: precursor ion mass tolerance ±15ppm; MS/MS tolerance ±20 mmu; two missed cleavages were allowed with the enzyme trypsin; carbamidomethylation (C) was set as fixed modification, and oxidation (M) and iTRAQ labeling (K, Y, and N-term) were set as dynamic modifications; peptides with peptide score ≥ 10 and false discovery rate (FDR) < 0.01. The protein identification contained at least one unique peptide. The proteome data was uploaded to the public repository iProX (ID: IPX0001238000).

### Bioinformatics analysis

GO annotation analysis, including molecular function, cellular component, and biological process of the differentially expressed proteins, was performed using Uniprot (http://www.uniprot.org/). The KEGG pathway database (http://www.genome.jp/kegg/) was used to classify and group these differentially expressed proteins (Kanehisa et al., 2007). KEGG pathway and GO enrichment analysis of the differentially expressed proteins were performed, and the formula used was:

$$\begin{array}{l} P=1-\overset{m-1}{\underset{i=0}{\sum }}\frac{\left(\begin{array}{c} M\\ i \end{array}\right)\left(\begin{array}{c} N-m\\ n-i \end{array}\right)}{\left(\begin{array}{c} N\\ n \end{array}\right)} \end{array}$$

where *N* represents the number of all identified proteins with a GO or a KEGG pathway annotation; *n* is the number of differential proteins in *N*; *M* is the number of proteins that are annotated to the specific GO term or pathway; and m is the number of differential proteins in *M*. If *p*-value is below 0.05, the GO term or pathway was defined as a significant enrichment of differential proteins. The false discovery rate (FDR) was controlled by the Bonferroni step-down test to correct the *p*-value.

### Real-time quantitative PCR

Quantitative real-time PCR primer pairs were designed using Primer 5.0 and primer sequences are listed in Table S1. Total RNA was extracted using a TRIzol kit (Invitrogen, USA) according to the manufacturer's instructions. The quantity of total RNA was measured using NanoDrop 2000 (Thermo Fisher Scientific), and the quality was assessed using 1.0% denaturing agarose gel electrophoresis. Complementary DNA was synthesized from 1000 ng RNA using PrimeScript RT reagent Kit with gDNA Eraser (Takara, Dalian, China). Quantitative real-time PCR was performed using the SYBR Premix Ex Taq kit (Tli RNaseH Plus) (Takara, Dalian, China) according to the manufacturer's instructions with the ABI 7500 Real Time PCR thermal cycler (Applied Biosystems) using the following cycle conditions: 95°C for 30 s, then 40 cycles at 95°C for 5 s and 60°C for 34 s, a final cycle of 95°C for 15 s, 60°C for 60 s, and 95°C for 15 s. Housekeeping genes, *β-actin* and 16S rRNA, were selected as reference genes to normalize the expression level of target genes using 2^−ΔΔ*CT*^ method (Livak and Schmittgen, 2001). All experiments were carried out using three biological replications and three technical replications. Differences in transcript expression in different tissues and developmental stages were analyzed with a one-way analysis of variance (ANOVA) using SPSS software (version 19.0; IBM, Armonk, NY, USA) followed by the least-significant difference (LSD) test. Differences were considered statistically significant at a *p*-value less than 0.05.

## Results

### Identification and quantification of differentially expressed proteins between alate and apterous morphs of *A. pisum*

**Figure 2:**
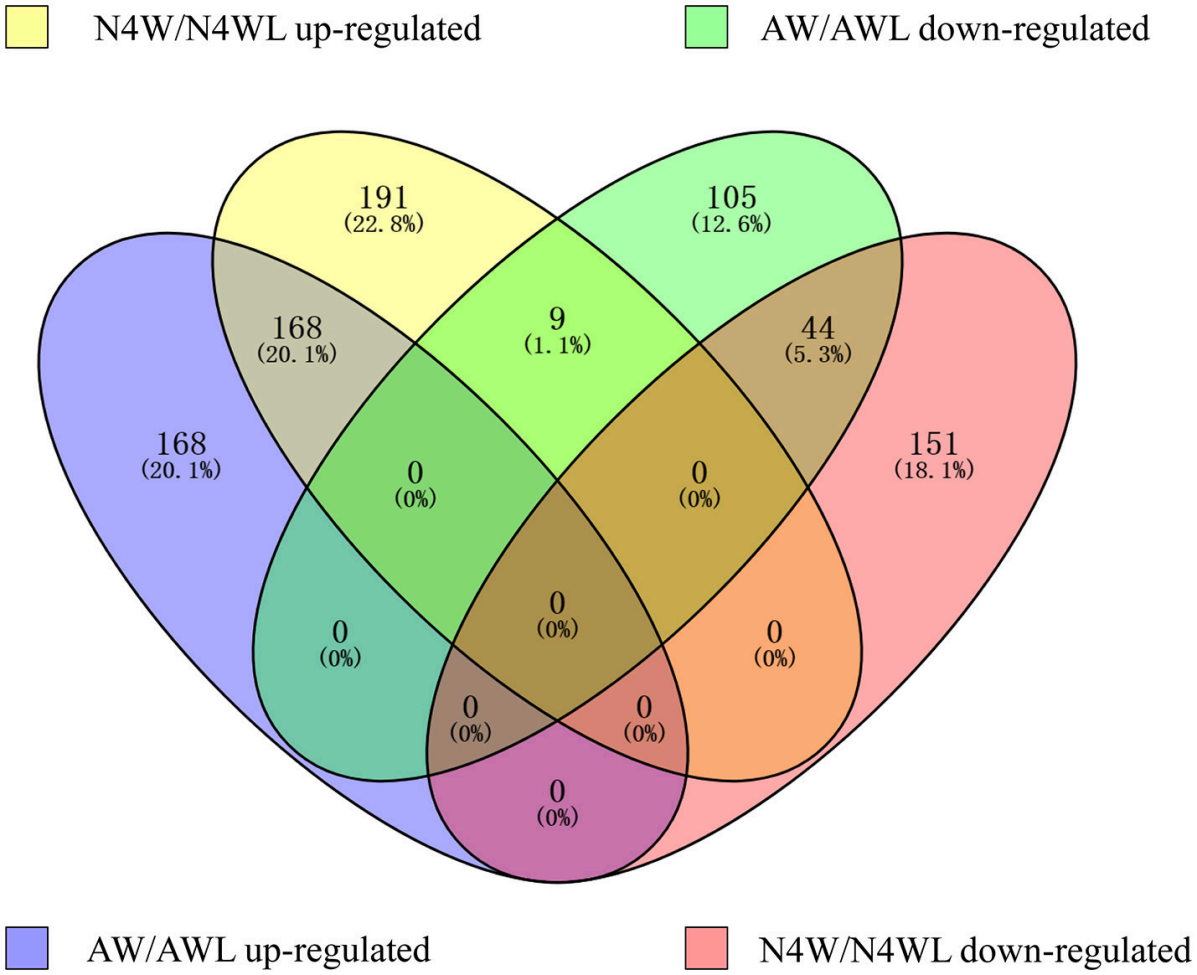
Venn diagram showing the distribution of 836 differentially abundant protein groups. AW, winged adults; AWL, wingless adults; N4W, winged fourth instar nymphs; N4WL, wingless fourth instar nymphs.

To investigate the differentially expressed proteins in alate and apterous morphs of *A. pisum*, quantitative iTRAQ labeling-based proteomic analysis was performed. A schematic representation of the experimental workflow is shown in Figure 1. Based on 4-plex iTRAQ proteomic labeling and LC-MS/MS analysis, a total of 5,116 protein groups were identified quantified in all experiments at the two developmental stages and three replicates (Table S2). Based on previous studies (Yang et al., 2013; Chen et al., 2016), DEPGs are defined based on a 1.2–1.5-fold change threshold. Among those proteins, only one protein in two or three replicates with fold changes ≥1.2 was defined up-regulated, or ≤0.83 was down-regulated. Following this criterion, a total of 563 DEPGs in alate fourth instar nymphs and 494 DEPGs in alate adults were detected, respectively. A total of 538 DEPGs were up-regulated under at least one developmental stage, including 168 DEPGs up-regulated at two stages (N4W/N4WL and AW/AWL), and 200 and 168 DEPGs up-regulated at N4W/N4WL and AW/AWL, respectively. Among 309 down-regulated DEPGs under at least one development stage, 44 DEPGs were shared by two stages (AW/AWL and N4W/N4WL), whereas 149 and 114 DEPGs were down-regulated at N4W/N4WL and AW/AWL, respectively (Figure 2).

In general, three clearly different expression profiles under different wing morphs at fourth instar nymph and adult stages were generalized among 836 DEPGs and the summarized data of DEPGs are listed in Tables S3–S5: 168 DEPGs (20.1%) at two stages were up-regulated; 44 DEPGs (5.3%) at two stages were down-regulated; 9 DEPGs (1.1%) were up-regulated in N4W then down-regulated in AW (Table S3); 151 DEPGs (18.1%) were down-regulated and 191 DEPGs (22.8%) were up-regulated only in the N4W sample (Table S4), whereas 105 DEPGs (12.6%) were down-regulated and 168 DEPGs (20.1%) were up-regulated only in the AW sample (Table S5).

### Functional enrichment analysis of differentially expressed proteins

**Figure 3:**
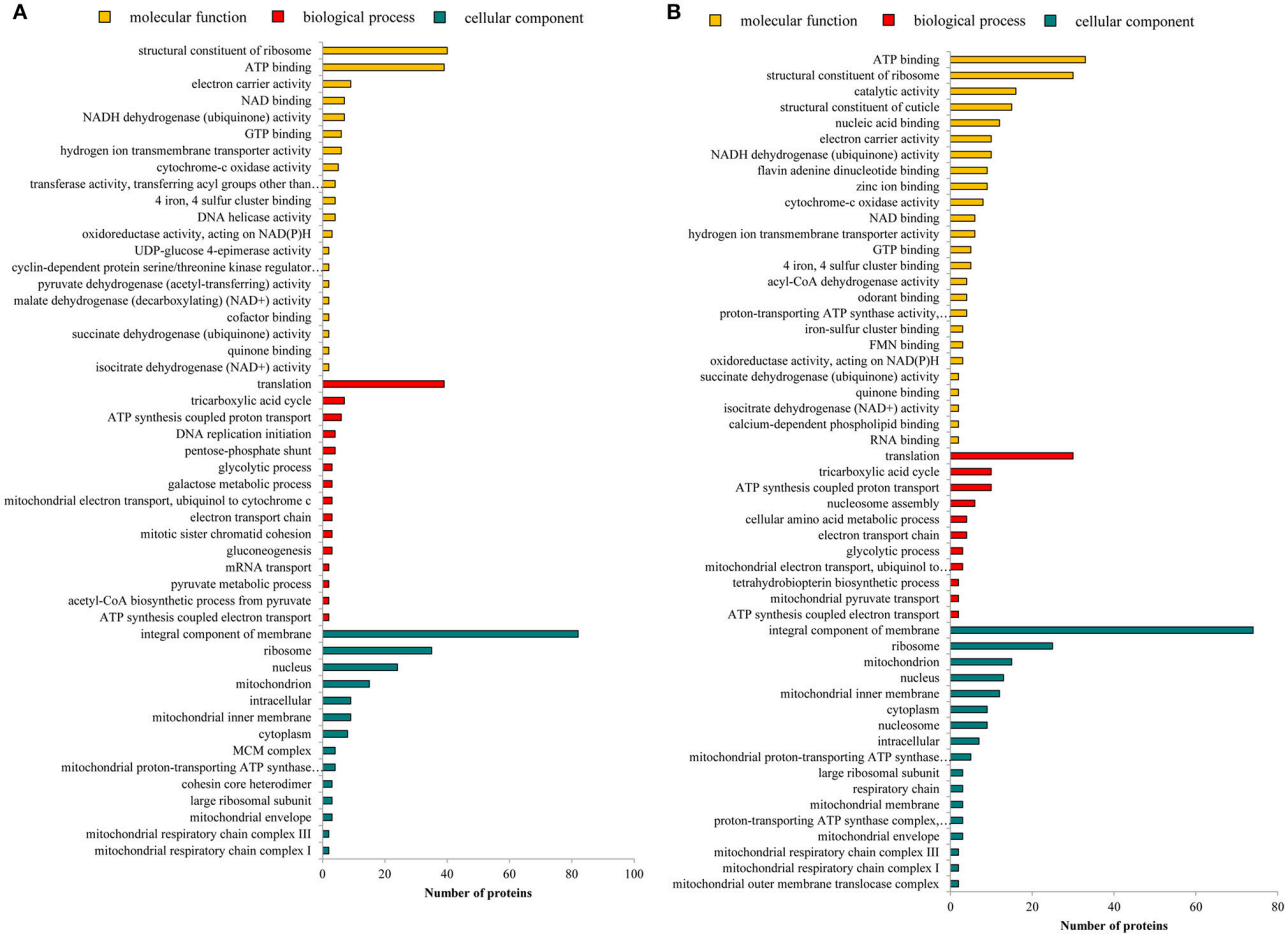
Enriched GO of differentially abundant protein groups in **(A)** N4W/N4WL and **(B)** AW/AWL from three categories: biological process, molecular function, and cellular component.

To analyze the differentially abundant protein groups between wingless and winged aphids, all DEPGs were submitted to Uniprot for functional annotation and a GO category enrichment analysis was conducted. The GO annotation of proteins included biological process, molecular function, and cellular component and the detailed information is shown in Figure 3. For molecular function, the differentially abundant proteins of N4W/N4WL and AW/AWL were mainly enriched in structural constituents of ribosome and adenosine triphosphate (ATP)-binding proteins. In addition, the DEPGs of AW/AWL were found enriched in odorant-binding. According to biological process, the differentially abundant protein groups between alate and apterous morphs were mainly assigned to translation and tricarboxylic acid cycle. The cellular component of DEPGs was categorized as the integral components of membrane, ribosome, nucleus, and mitochondrion.

**Figure 4:**
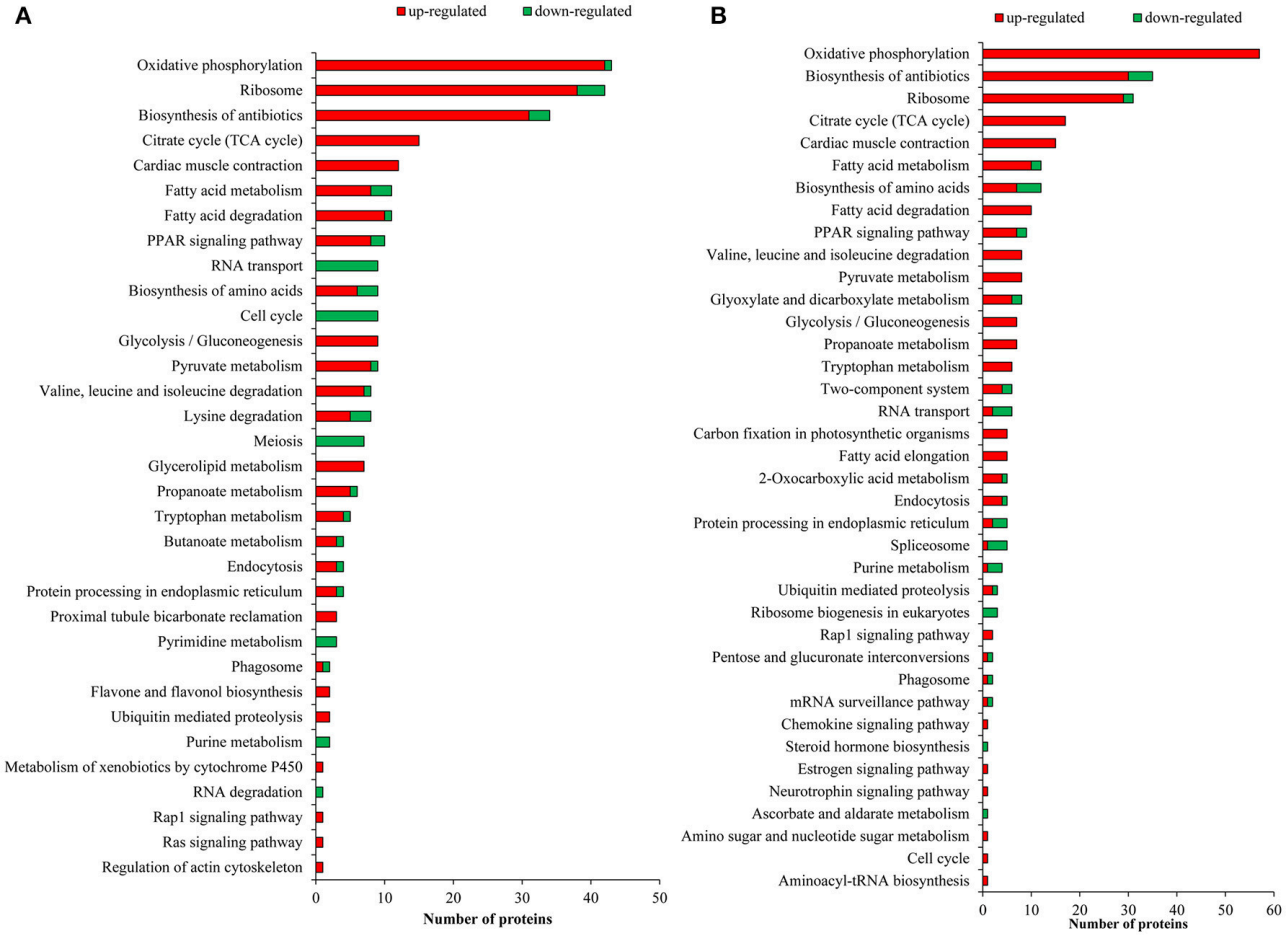
Enriched Kyoto Encyclopedia of Genes and Genomes (KEGG) pathways of differentially abundant protein groups in **(A)** N4W/N4WL and **(B)** AW/AWL. Green and red bars represent down-regulated and up-regulated proteins in alate morphs, respectively.

**Figure 5:**
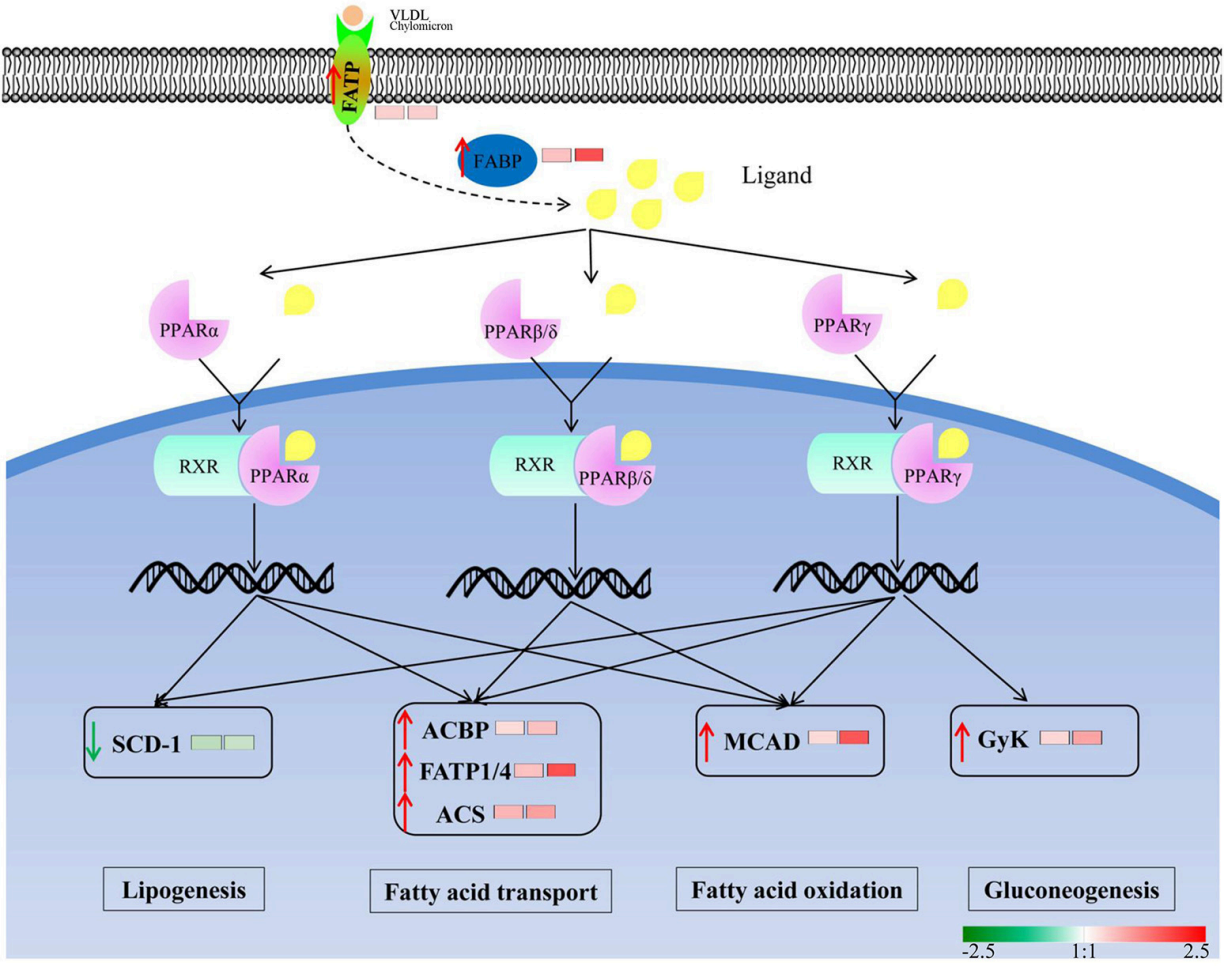
Differentially abundant protein groups between winged and wingless aphids are involved in Peroxisome proliferator-activated receptor (PPAR) signal pathway in KEGG. Small molecules ligands bind to PPARs and forms a heterodimer with RXR, then induces conformational changes in PPARs that lead to their transcriptional activation/modulation to facilitate lipid metabolism and gluconeogenesis. Green and red arrows represent down-regulated and up-regulated proteins in alate morphs, respectively. The left and right squares represent the fold change in winged fourth instar nymphs and winged adults, respectively. The abbreviations were noted as follows: FATP, fatty acid transport protein; FABP, fatty acid binding protein; MPA13 allergen; ACBP, acyl-CoA-binding protein; GyK, glycerol kinase; ACS, long-chain-fatty-acid–CoA ligase; MCAD, medium-chain specific acyl-CoA dehydrogenase; SCD-1,stearoyl-CoA desaturase.

To investigate the enrichment pathways of the DEPGs between winged and wingless morphs of aphids, KEGG analysis was performed. According to KEGG analysis, 33 pathways were enriched (*p*-value ≤ 0.05) in N4W/N4WL and the main KEGG functional classifications were oxidative phosphorylation, ribosome, biosynthesis of antibiotics, tricarboxylic acid (TCA) cycle, cardiac muscle contraction, fatty acid degradation, fatty acid metabolism, peroxisome proliferator-activated receptor (PPAR) signaling pathway, pyruvate metabolism, and glycolysis/gluconeogenesis (Figure 4A). In AW/AWL, 38 pathways were enriched (*p*-value ≤ 0.05) and the main KEGG functional classifications of the DEPGs were oxidative phosphorylation, biosynthesis of antibiotics, ribosome, citrate cycle (TCA cycle), cardiac muscle contraction, biosynthesis of amino acids, fatty acid metabolism, fatty acid degradation, PPAR signaling pathway, glyoxylate and dicarboxylate metabolism, and pyruvate metabolism (Figure 4B). DEPGs between alate and apterous morphs involved in the PPAR signal pathway in KEGG is shown in Figure 5 and DEPGs in this pathway were listed in Table S6.

### Transcriptional expression analysis of selected proteins as revealed by qRT-PCR

To evaluate the proteomic data and provide further information of the correlation between protein abundance and their mRNA expression patterns, qRT-PCR was performed to quantify the mRNA transcript level for 29 proteins including top 19 up-regulations in winged adult and top 10 up-regulations in wingless adult. The result showed that expression profiles of 26 genes were consistent with the proteomic changes and the mRNA levels (Table 1).

**Table 1:** qRT-PCR was performed to quantify the mRNA transcript level for 29 proteins including 19 up-regulations and 10 down-regulations in winged adult.

**Accession**	**Description**	**Score -R1**	**Score -R2**	**Score -R3**	**Coverage -R1**	**Coverage -R2**	**Coverage -R3**	**#Unique Peptides -R1**	**#Unique Peptides -R2**	**#Unique Peptides -R3**	**Fold change in DEPG**			**Fold change in qRT-PCR**
											**R1**	**R2**	**R3**	
641651025	PREDICTED: phosphatidylinositol 3-kinase regulatory subunit gamma-like	0.00	0.00		1.38	1.38		1	1		5.64	2.42	NA	0.57
328708637	PREDICTED: glutaredoxin domain-containing cysteine-rich protein CG12206-like	0.00		0.00	1.44		1.44	1		1	2.61	NA	2.97	0.51
328718811	PREDICTED: histone H1A, sperm-like	333.19	264.83	386.83	44.12	35.78	48.53	1	1	9	2.74	2.78	1.57	0.19
**240849049**	**acylphosphatase, putative**	**38.83**	**35.96**	**47.20**	**7.84**	**18.63**	**16.67**	**1**	**2**	**2**	**3.07**	**4.26**	**4.11**	**40.04**
**193699891**	**PREDICTED: phosphate carrier protein, mitochondrial-like**	**150.17**	**112.42**	**101.83**	**27.14**	**29.79**	**26.25**	**8**	**9**	**9**	**3.39**	**3.00**	**2.50**	**33.51**
**193573506**	**PREDICTED: flightin**	**72.62**	**84.33**	**59.96**	**25.63**	**25.13**	**25.63**	**5**	**4**	**5**	**1.97**	**2.92**	**3.62**	**2835.00**
**328722469**	**PREDICTED: DNA ligase 1-like**	**45.42**	**67.90**	**110.34**	**8.05**	**5.54**	**10.07**	**5**	**3**	**6**	**1.43**	**2.97**	**3.80**	**15.38**
**240848967**	**adenine nucleotide translocator-like**	**975.68**	**906.42**	**883.22**	**39.35**	**44.19**	**51.61**	**11**	**13**	**14**	**2.82**	**2.41**	**2.55**	**32.75**
**641658576**	**PREDICTED: protein 5NUC**	**33.16**	**37.72**	**45.33**	**1.34**	**7.16**	**6.42**	**1**	**4**	**4**	**2.63**	**2.15**	**2.47**	**1.17**
**193629643**	**PREDICTED: probable pyruvate dehydrogenase E1 component subunit alpha, mitochondrial**	**363.23**	**260.54**	**261.70**	**18.73**	**18.73**	**15.19**	**4**	**4**	**3**	**2.07**	**2.94**	**1.92**	**6.58**
**641661277**	**PREDICTED: 4-coumarate–CoA ligase 3-like**	**240.76**	**331.60**	**279.41**	**23.57**	**26.84**	**20.62**	**14**	**13**	**11**	**2.01**	**2.27**	**2.14**	**21.57**
**641677824**	**PREDICTED: short-chain specific acyl-CoA dehydrogenase, mitochondrial**	**109.02**	**164.49**	**138.82**	**14.11**	**19.95**	**18.73**	**5**	**7**	**6**	**2.17**	**2.01**	**2.17**	**3.65**
**193662009**	**PREDICTED: protein CREG1-like**	**49.09**	**67.51**	**84.78**	**7.63**	**10.59**	**7.63**	**2**	**3**	**2**	**2.00**	**2.19**	**2.15**	**3.64**
**328724202**	**PREDICTED: succinyl-CoA ligase [ADP-forming] subunit beta, mitochondrial**	**376.88**	**170.87**	**175.97**	**18.85**	**16.80**	**19.26**	**10**	**8**	**8**	**2.20**	**2.04**	**2.01**	**1.54**
**240849479**	**MPA13 allergen-like**	**488.31**	**619.05**	**434.88**	**37.78**	**51.11**	**45.19**	**6**	**8**	**7**	**2.09**	**2.09**	**1.90**	**2.23**
**328716751**	**PREDICTED: cytochrome c oxidase subunit 7A-related protein, mitochondrial-like**	**40.07**	**31.44**	**31.87**	**10.71**	**10.71**	**10.71**	**1**	**1**	**1**	**1.75**	**2.00**	**2.27**	**1.51**
**193706910**	**PREDICTED: probable medium-chain specific acyl-CoA dehydrogenase, mitochondrial**	**232.78**	**126.72**	**165.63**	**27.21**	**25.30**	**23.39**	**11**	**9**	**9**	**1.69**	**2.20**	**2.06**	**6.42**
**240848565**	**uncharacterized protein LOC100166422 precursor**	**61.32**	**69.14**	**34.44**	**13.13**	**18.75**	**16.25**	**1**	**2**	**1**	**2.18**	**2.70**	**1.00**	**4.34**
**193594238**	**PREDICTED: probable isocitrate dehydrogenase [NAD] subunit alpha, mitochondrial**	**495.86**	**270.95**	**294.85**	**27.93**	**28.21**	**28.21**	**11**	**11**	**11**	**2.29**	**1.48**	**2.03**	**1.63**
**193634238**	**PREDICTED: flocculation protein FLO11-like**	**148.28**	**192.58**	**158.73**	**9.48**	**11.74**	**10.05**	**9**	**10**	**8**	**0.85**	**0.68**	**0.68**	**0.06**
**240848949**	**cuticular protein 11 precursor**	**44.90**	**88.42**	**84.67**	**11.30**	**44.35**	**26.09**	**1**	**3**	**2**	**0.87**	**0.69**	**0.64**	**0.14**
**328724839**	**PREDICTED: thymidylate kinase**	**38.01**	**32.31**		**9.43**	**5.19**	**1**	**2**	**1**	**NA**	**0.68**	**0.68**	**0.49**
**253735780**	**cuticle protein-like precursor**	**42.91**	**51.11**	**19.29**	**12.82**	**12.82**	**12.82**	**2**	**2**	**2**	**0.75**	**0.60**	**0.69**	**0.34**
**193706873**	**PREDICTED: tyrosine-protein phosphatase non-receptor type 23-like**	**1323.41**	**1438.25**	**846.73**	**30.94**	**29.43**	**34.45**	**2**	**2**	**2**	**0.79**	**0.67**	**0.59**	**0.89**
**242247276**	**acireductone dioxygenase-like**	**126.17**	**94.83**	**132.31**	**22.10**	**16.02**	**17.13**	**5**	**3**	**4**	**0.59**	**0.76**	**0.67**	**0.29**
**641661105**	**PREDICTED: peroxidasin homolog**	**37.35**	**42.40**	**47.93**	**5.57**	**3.21**	**7.71**	**2**	**1**	**1**	**0.61**	**0.66**	**0.68**	**0.05**
**240849581**	**cuticular protein 10 precursor**	**233.94**	**322.95**	**223.93**	**42.86**	**37.86**	**37.86**	**3**	**3**	**3**	**0.59**	**0.57**	**0.57**	**0.07**
**328721916**	**PREDICTED: fibril-forming collagen alpha chain-like**		**0.00**	**53.21**		**0.68**	**0.68**	**1**	**1**	**1**	**NA**	**0.41**	**0.69**	**0.03**
**229892236**	**odorant-binding protein 4 precursor**	**34.28**	**24.51**	**65.00**	**13.47**	**7.77**	**11.40**	**2**	**1**	**2**	**0.51**	**0.23**	**0.31**	**0.30**

*Bold values mean that transcriptional expression of 26 genes candidates of differentially expressed proteins were consistent with those at proteomic levels*.

**Figure 6:**
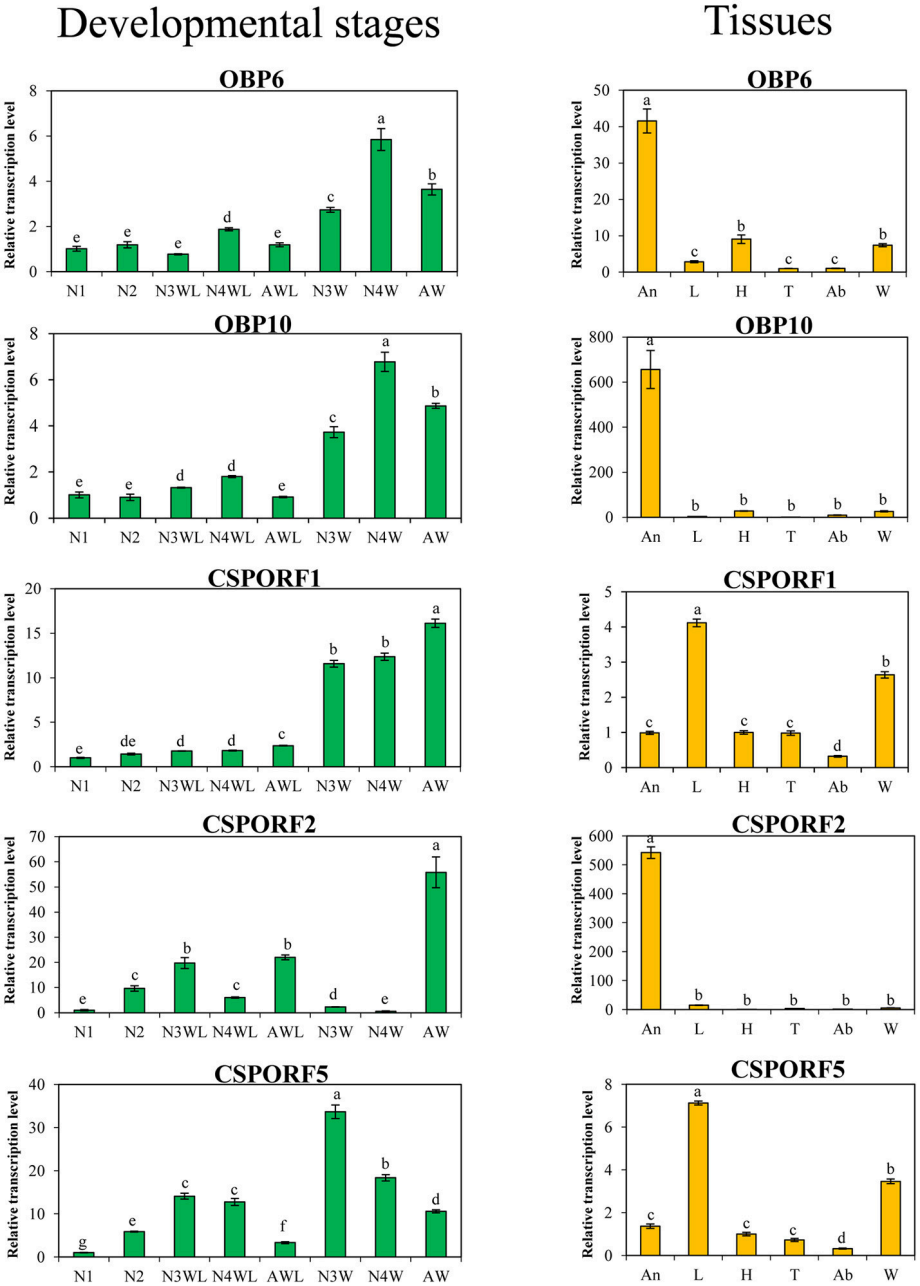
Relative expression levels of higher expressed OBPs and CSPs in alate morphs in different developmental stages (green) or different body parts (orange) of *A. pisum*. N1, first instar nymphs; N2, second instar nymphs; N3WL, wingless third instar nymphs; N4WL, wingless fourth instar nymphs; AWL, wingless adults; N3W, winged third instar nymphs; N4W, winged fourth instar nymphs; AW, winged adults; An, antennae; L, legs; H, heads; T, thoraxes; Ab, abdomens; W, wings. Lowercase letter above each bar indicates a significant difference (*P* < 0.05) in mean transcript levels which were compared using one-way ANOVA, followed by the least-significant difference (LSD) method.

According to GO analysis, the DEPGs of AW/AWL were enriched in odorant-binding. The description and fold changes of four chemosensory proteins (CSPs) and four odorant-binding proteins (OBPs) that were differentially expressed between alate and apterous morphs are summarized in Table 2. qRT-PCR was performed to quantify the mRNA transcript level for them. In addition, the expression patterns of OBP3, OBP6 to OBP13 between alate and apterous *A. pisum* adults were investigated at mRNA levels (Figure S1). The results showed that OBP6 and OBP10 were significantly up-regulated in alate adults. Based on combined analyses of proteomics and mRNA expression profiles, up-regulated genes in alate morph aphids including three CSPs and two OBPs were further investigated in different body parts, instars, and wing morphs (Figure 6). Compared with apterous adults, transcriptional expression levels of three CSPs were significantly higher in alate adults. The results showed similar expression patterns as indicated by the proteomics analysis. CSPORF1 genes were sharply increased from the third instar nymphs to adults of alate morphs. An expression peak was present in the alate adults and apterous third instar nymphs, for CSPORF2 and CSPORF5 genes, respectively. The transcriptional expression profiles of OBP6 and OBP10 in alate aphids shared the similar patterns with CSPORF1. Transcription profiles for these genes were also determined in different body parts. For OBP6, OBP10, and CSPORF2, the highest transcript levels were observed in the antennae. The expression levels of CSPORF1 and CSPORF5 were both significantly higher in the legs, followed by high expression in the wings.

**Table 2:** OBP and CSP genes differentially expressed between alate and apterous samples.

**Accession**	**Description**	**Abbreviation**	**Score -R1**	**Score -R2**	**Score -R3**	**Coverage -R1**	**Coverage -R2**	**Coverage -R3**	**# Unique Peptides -R1**	**# Unique Peptides -R2**	**# Unique Peptides -R3**	**Fold change in AW/AWL**			**Fold change in qRT-PCR**
												**-R1**	**-R2**	**-R3**	
187125206	chemosensory protein-like precursor	CSPORF1	152.31	162.97	126.19	37.24	45.52	45.52	6	6	7	1.53	1.67	1.64	6.805
187125204	chemosensory protein-like precursor	CSPORF2	85.34	56.46	62.67	6.11	17.56	13.74	2	4	2	1.39	1.10	1.24	2.535
187123200	chemosensory protein-like precursor	CSPORF5	223.24	133.56	165.80	40.15	40.15	39.42	5	5	5	1.33	1.36	1.20	3.187
242247533	chemosensory protein 1-like precursor	CSP1L	0.00	22.99		5.16	5.16		1	1		0.72	0.70	NA	0.576
229892228	odorant-binding protein 1 precursor	OBP1	125.58	52.00	79.65	20.13	20.13	20.13	4	3	4	0.76	0.89	0.70	0.545
229892232	odorant-binding protein 2 precursor	OBP2	52.56	0.00	33.53	6.17	3.29	3.29	2	1	1	0.97	0.74	0.67	**0.98**
229892236	odorant-binding protein 4 precursor	OBP4	34.28	24.51	65.00	13.47	7.77	11.40	2	1	2	0.51	0.23	0.31	0.314
229892238	odorant-binding protein 5 precursor	OBP5	97.50	81.99	63.58	13.12	13.12	8.60	3	3	2	0.80	0.91	0.70	0.35

*AW, winged adults; AWL, wingless adults*.

## Discussion

Flight capability is a vital feature in insects and plays an important role in their evolutionary success. Flight benefits dispersal capacity balanced against potential metabolic, reproductive, and survival costs (Langellotto et al., 2000; Castañeda et al., 2010). Here, iTRAQ-coupled 2D LCMS/MS was used to analyze the molecular mechanisms of wing development and dimorphism in *A. pisum*. By analyzing the differential expression of proteins between alate and apterous aphids at the fourth instar nymph and adult stages, a total of 836 DEPGs were obtained between alate and apterous morph aphids, of which 538 DEPGs were up-regulated under at least one developmental stage, whereas 309 were down-regulated. In both stages, most of the differentially expressed proteins showed higher levels in alate morphs than in apterous morphs. Those genes associated with flight capability are more than those associated with reproduction and this discrepancy is a reflection of trade-offs between dispersal capability and reproductive structures. The number of DEPGs between winged fourth instar nymphs and winged adults were similar.

### Differentially expressed proteins involved in energy metabolism

The trade-offs between flight capability and other traits result in energy allocation discrepancy between alate and apterous morphs (Roff and Fairbairn, 1991; Zera et al., 1998). In this study, DEPGs of different morphs mainly participated in energy metabolism pathways including oxidative phosphorylation (path: ko00190), citrate cycle (TCA cycle) (path: ko00020), fatty acid metabolism (path: ko01212), fatty acid degradation (path: ko00071), glycolysis/gluconeogenesis (path: ko00010), pyruvate metabolism (path: ko00620), and propanoate metabolism (path: ko00640). These results reflected active and complex protein abundance change patterns between alate and apterous aphids at the molecular level. Differential energy allocation is a crucial part of trade-offs between dispersal capability and reproduction, with alate morphs investing energy into building wings and flight muscles to maintain energetically costly flight performance rather than developing quickly and maintaining high levels of offspring production as in apterous morphs (Brisson, 2010). Most DEPGs were related to energy metabolism and showed significantly up-regulated expression levels in alates (Figure 4), which suggested that winged morphs require more energy and have higher metabolic costs than wingless morphs. In flying, organisms incur two costs: developing a flight apparatus and fueling for flight (Dixon and Kindlmann, 1999). The results showed that up-regulated proteins involved in energy production at fourth instar nymph and adult stages were different, and these results agree with the metabolic requirements for the construction of wings and muscles as fourth instars and the capability of flight and the maintenance of flight muscles as adults (Zera and Denno, 1997).

Our research suggested that lipids provide resources for wing development and dispersion of alate aphids. Lipids are mainly stored in the fat body of insects as triacylglycerol and are used as fuel in flight muscles (Chino and Downer, 1982). The content of triacylglycerol is higher in alate morphs of aphids (Dixon et al., 1993; Itoyama et al., 2000; Xu et al., 2011), which is the same situation as in long-winged planthoppers (Itoyama et al., 1999) and crickets (Zera et al., 1994; Zera and Larsen, 2001). Correspondingly, well-developed flight muscles are reported for winged aphids (Ishikawa and Miura, 2007), long-winged crickets (Mole and Zera, 1993; Tanaka, 1993; Zera et al., 1997), and firebugs (Socha, 2006). In our study, the three proteins that showed significantly higher expression levels in alate adults associated with fatty acid metabolism and degradation were short-chain specific acyl-CoA dehydrogenase (SCAD), medium-chain specific acyl-CoA dehydrogenase (MCAD), and isocitrate dehydrogenase (NAD) subunit alpha (IDH3A) (Table S5). MCAD catalyzes the initial step of fatty acid beta-oxidation, and SCAD is a key enzyme of fatty acid β-oxidation. This result is consistent with the previous observation that the content of total lipid, triglyceride, and free fatty acid was dramatically higher in winged adults (Beenakkers et al., 1985; Itoyama et al., 2000; Shi et al., 2010). In apterous adults, only fatty acid desaturase-like (FADS) and stearoyl-CoA desaturase-like (SCD) related to fatty acid metabolism were up-regulated.

Glycogen also provides resources for alate aphids besides lipids, which is consistent with the report in alate brown citrus aphid, *Toxoptera citricida* (Shang et al., 2016). Early reports showed that both lipid and glycogen are consumed during tethered flight of insects such as migratory locust (*Locusta migratoria*) (Worm and Beenakkers, 1980), planthopper (*Nilaparuara lugens*) (Padgham, 1983), and *Aphis fabae* Scop (Cockbain, 1961). Glycogen is used during early flight, and fat is the principal fuel after the first hour (Cockbain, 1961). Pyruvate is a key intersection in the network of metabolic pathways and is known as the “hub” of carbohydrate, fatty acids, and proteins (Tatusov et al., 2003; Simpson et al., 2011). Pyruvate can be made from glucose through glycolysis (Simpson et al., 2011). In this study, DEPGs that were involved in glycolysis/gluconeogenesis and pyruvate metabolism were almost up-regulated in alate morphs aphids. However, these genes were different between winged fourth instars and winged adults. Pyruvate dehydrogenase E1 component subunit alpha (PDHA1) gene, with higher expressions in winged fourth instars and adults (Table S3), was involved in pyruvate metabolism, whereas acylphosphatase gene only had higher expression in alate adults. Our results suggested glycogen and lipid not only provide energy resources for flight in adults but also for development of wing and muscle of fourth instars (Shang et al., 2016).

### Differentially expressed proteins involved in amino acid biosynthesis and metabolism

In this study, DEPGs mainly participated in amino acid biosynthesis and metabolism, including ribosome (path: ko03010); biosynthesis of amino acids (path: ko01230); valine, leucine, and isoleucine degradation (path: ko00280); tryptophan metabolism (path: ko00380); spliceosome (path: ko03040); and RNA transport (path: ko03013). The ribosome is required for protein synthesis and plays vital roles in the growth and development of organisms (Zhu et al., 2017). Early reports showed gene products that are components of ribosomes were over-represented in *A*. *pisum* (Brisson et al., 2007). Our study found that more ribosomal protein was detected in the alates, which implied that ribosomal proteins play important roles in development and dispersal of winged aphids. Many proteins in the ribosome were differentially expressed between alates and apterous aphids, including neurofilament heavy polypeptide and ribosomal protein small and large subunits. Ribosomal protein L19e-like, peptidyl-prolyl cis-trans isomerase-like, and 40S ribosomal protein S21-like, which were highly expressed in wingless fourth instars and adults (Tables S3, S4) might be also important for the development and reproduction of wingless morphs (Xue et al., 2010). The functions and mechanisms of ribosomal proteins between the two morphs remain largely unknown, and our data of ribosome proteins expression profiles in the pea aphid can assist in understanding them in future.

### Differentially expressed proteins involved in signal sensing and transduction

In this study, we found that PPAR signaling pathway (path: ko03320) (Figure 5), which is thought to participate in lipid metabolism (Schoonjans et al., 1996), was the co-enriched pathway at alate fourth instar nymphs and adults and might play a critical role in the wing development and dispersal of aphids. Early reports found that PPAR signaling pathway was significantly up-regulated in dispersing morphs (Xue et al., 2010; Shang et al., 2016). In the current study, most DEPGs in this pathway were up-regulated in alates (Table S6) and are involved in facilitating lipid metabolism and gluconeogenesis to increase metabolism of energy sources. Fatty acid binding protein (FABP), which is a small cytosolic protein abundantly found in the muscle and transports lipophilic molecules from the outer cell membrane to certain intracellular receptors, exhibits significantly higher expression in alate morphs (Tan et al., 2002). Fatty acid transport proteins (FATPs) are a family of six integral membrane proteins with an extracellular/luminal N-terminal and C-terminal domain with fatty acyl-CoA synthetase activity. In the future, to reveal the mechanism of wing development of aphids, more genes and proteins including those in the PPAR-related metabolic pathways require further study.

For flight navigation and detecting new habitats, alate aphids have a more detailed sensory system (Tsuji and Kawada, 1987b; Hazell et al., 2005). In this study, proteins involved in chemoreception are also significantly different between winged and wingless morphs. Early reports suggested that alarm pheromone, a volatile compound released from aphid colonies' cornicles due to high-density triggers or predator attacks, could induce aphids to produce winged dispersal morphs (Kunert et al., 2005; Verheggen et al., 2009; Hatano et al., 2010). Studies showed that an unidentified “spacing pheromone” released from crowded aphids could change their behaviors (Pettersson et al., 1995). In some aphid species, antennae act as a pivotal part in the perception of tactile signals (Johnson, 1965; Lees, 1967; Sutherland, 1969). OBPs are small, water-soluble proteins abundant in sensillar lymph of insect antennae and other non-sensory organs that transport hydrophobic semiochemicals (pheromones and plant volatiles) through the sensillar lymph and finally reach sensory dendrites, where released chemicals activate membrane-bound odorant receptors (Brito et al., 2016). Like OBPs, CSPs belong to another family of small, soluble proteins (Brito et al., 2016). The abundance and diverse expression patterns of different CSPs suggest that they are involved in multiple functions in insects such as recognition of sex pheromones (Jacquin-Joly et al., 2001) and general odorants (Liu et al., 2012), development (Maleszka et al., 2007), and feeding (Liu et al., 2014). Based on combined analysis of transcriptome and proteome, higher expression levels of two OBP and three CSP genes in alate morphs were investigated. OBP6 and OBP10 were of higher expression in alate aphids, suggesting a possible role in wing development and migration. OBP6 had significantly higher expression in antennae in alate morphs, suggesting an olfactory role for this protein in discrimination (*E*)-β-farnesene, “spacing pheromone” or mediating the perception of molecules related to new host-plant location (Vogt et al., 1999; Sun et al., 2012; De Biasio et al., 2015; Xue et al., 2016). The expression pattern of OBP10 is similar to OBP6, which might have similar function. In addition, OBP6 was also abundantly expressed in heads (without antennae) of alate adults, suggesting a possible role in host-plant selection during migration (De Biasio et al., 2015). Besides OBPs, abundant expression of three CSP genes was also detected in alate morphs. CSPORF1 and CSPORF5, which were abundantly expressed in legs, followed by high expression in the wings of winged aphids, might be involved in contact with the plant, leaf surface characteristics, or the process of volatile reception and be indicative of mechanoreceptor or chemoreception sensilla on the legs and wings (Pettersson et al., 2007; Zhou et al., 2008; Yasukawa et al., 2010; Harada et al., 2012). CSPORF2, which is specifically expressed in antennae and only increased in winged adults, might be involved in chemoreception during migration (Ghanim et al., 2006; González et al., 2009; Xue et al., 2016). RNA interference (RNAi) and fluorescence competition assays should be used in the future to investigate the function of these genes.

## Conclusions

In conclusion, this study is the first report to investigate protein expression profiles between winged and wingless aphids using a new proteomic profiling method iTRAQ-coupled 2D LC-MS/MS. A total of 836 differentially expressed protein groups were detected, in which 563 and 494 DEPGs were identified in alate aphids at fourth instar nymph and adult stages, respectively. Based on the GO and KEGG enrichment analysis, we concluded that olfactory senses have an important function in alate aphids and winged aphids using lipids and glycogen as fuel resources for wing development and migration. In addition, protein groups involved in the PPAR signaling pathway of aphids were found to play a crucial role in winged morphs. Although our report provides knowledge of some proteins associated with development and dispersion, gene function analysis is needed to further understand the roles of these proteins. Our findings may provide new clues for elucidating the molecular mechanisms underlying wing dimorphism in aphids.

## Author contributions

LS, YG, and LB designed the experiments. LS, YG, and JL preformed the experiments. LS and LB analyzed data and drafted the manuscript. LS, JL, and LB revised the manuscript. All authors read and approved the manuscript for final submission.

### Conflict of interest statement

The authors declare that the research was conducted in the absence of any commercial or financial relationships that could be construed as a potential conflict of interest.

## Supplementary material

Figure S1 Expression profiles of OBPs in adult of *Acyrthosiphon pisum* between alate and apterous morphs. Lowercase letter above each bar indicates a significant difference (*P* < 0.05) in mean transcript levels of alate *vs* apterous which were compared using one-way ANOVA, followed by the least-significant difference (LSD) method.

Table S1 Primers of quantitative RT-PCR for the selected genes.

Table S2 The proteins were identified and quantified.

Table S3 The DEPs were identified and quantified both in winged fourth instar-nymphs and winged adults.

Table S4 List of up- or down-regulated proteins under only winged morphs at fourth instar-nymph stage.

Table S5 List of up- or down-regulated proteins under only winged morphs at adult stage.

Table S6 DEPs involved in PPAR signal pathway at fourth instar-nymph and adult stage.
