# Supplementary figures and images for: iTRAQ-Based Comparative Proteomic Analysis Reveals Molecular Mechanisms Underlying Wing Dimorphism of the Pea Aphid, Acyrthosiphon pisum
Source: Front Physiol. 2018 Aug 7;9:1016. doi: 10.3389/fphys.2018.01016 (PMC6090017; doi:10.3389/fphys.2018.01016)

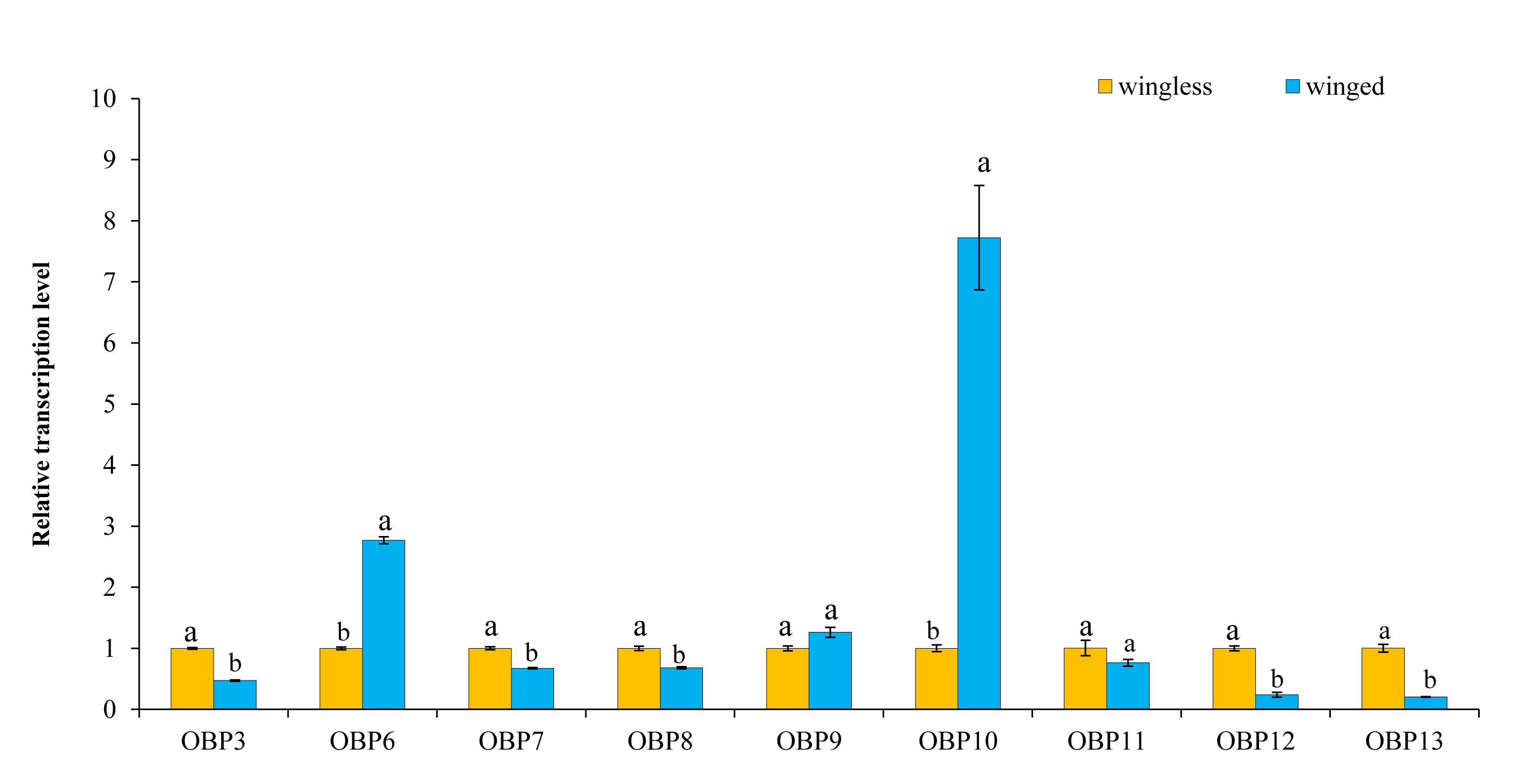

Supplement: Figure S1 — Expression profiles of OBPs in adult of Acyrthosiphon pisum between alate and apterous morphs. Lowercase letter above each bar indicates a significant difference (P < 0.05) in mean transcript levels of alate vs apterous which were compared using one-way ANOVA, followed by the least-significant difference (LSD) method. [file Image_1.TIF]
